# Association between anemia-related biomarkers and the adequacy of peritoneal dialysis in Chinese patients with chronic kidney disease

**DOI:** 10.3389/fphys.2023.1170537

**Published:** 2023-07-27

**Authors:** Jia-Lin Li, Zhen Cai, Jing Zhao, Xiang-Gang Zhu, Qian Li, Yan-Shuang Li, Meng-Chao Liu, Fang-Qiang Cui, Wen-Jing Zhao, Wen-Quan Niu

**Affiliations:** ^1^ Department of Nephropathy, Beijing Hospital of Traditional Chinese Medicine, Capital Medical University, Beijing, China; ^2^ Center for Evidence-Based Medicine, Capital Institute of Pediatrics, Beijing, China

**Keywords:** chronic kidney disease, peritoneal dialysis, Kt/V urea, anemia, nomogram

## Abstract

**Objectives:** The study aimed to examine the association of three anemia-related biomarkers with the adequacy of peritoneal dialysis (PD) in patients with chronic kidney disease (CKD).

**Methods:** This study included 127 PD patients. The total Kt/V urea (Kt/V) was calculated according to the Kidney Disease Outcomes Quality Initiative (K/DOQI) guidelines. All patients were classified into two groups based on Kt/V, viz., adequate (Kt/V ≥1.7) and inadequate (Kt/V <1.7) groups. Effect sizes are expressed as odds ratios (ORs) and 95% confidence interval (CI).

**Results:** After adjusting for age, gender, hypertension, diabetes, and PD duration, 20 g/L increment in hemoglobin (Hgb) was observed to significantly reduce the risk of inadequate PD by 19% (OR; 95% CI; P: 0.81; 0.70 to 0.95; 0.009), 5 g/L increment in the mean corpuscular hemoglobin concentration (MCHC) by 7% (0.93; 0.88 to 0.98; 0.009), and 5% increment in transferrin saturation (TS) by 23% (0.77; 0.64 to 0.94; 0.012). The gender-specific nomogram model was constructed by incorporating three significant anemia-related biomarkers and convenient influencing factors, and the prediction accuracy was good (concordance index (C-index): 0.686 for men and 0.825 for women).

**Conclusion:** Our findings indicate that the deterioration of three anemia-related biomarkers (Hgb, MCHC, and TS) can precipitate the development of inadequate PD in Chinese patients with CKD.

## Introduction

Chronic kidney disease (CKD) is a chronic disorder of public health importance affecting approximately 1/10 of adults worldwide (Yang et al., 2020a; Kovesdy, 2022). Echoing the high global burden of CKD, along comes kidney replacement therapy, which is expected to increase over the coming decade. Yet, due to the scarcity of organs for transplantation and healthcare cost of hemodialysis, as well as lack of hemodialysis centers, peritoneal dialysis (PD), a form of home dialysis, is gaining popularity. Global statistics reveal an estimated 272,000 PD patients, accounting for 11% of dialysis patients (Li et al., 2017; Bartosova and Schmitt, 2018; Yang et al., 2020b; Himmelfarb et al., 2020; Thurlow et al., 2021). In China, the number of PD patients in 2017 was 86,264, which increases annually (Wilkie and Davies, 2018; Yang et al., 2021).

In patients with CKD, PD can meet the requirements of clearing uremic toxins and reaching an acid–base balance, as well as removing excess fluids (Chan et al., 2019). Dialysis adequacy should be monitored at regular intervals. It is widely acknowledged that the urea clearance index, Kt/V urea (Kt/V), is a key index of dialysis adequacy, and its minimum target value is recommended to be 1.7 according to the Kidney Disease Outcomes Quality Initiative (K/DOQI) and the International Society for Peritoneal Dialysis (ISPD). The calculation of Kt/V, which requires 24-h peritoneal dialysate and 24-h urine samples, is usually impractical in outpatients, which inspires us to seek other reliable and convenient biomarkers that can be used as predictors for the dialysis adequacy status.

It is widely recognized that renal anemia is a common complication of uremic patients, and patients with a lower Kt/V level require more erythropoietin to manage anemia. Several studies showed that patients with the total Kt/V level maintained below 1.70 as a lower level in hemoglobin indicated inadequate PD (Lo et al., 2003; Liu et al., 2022). In patients with persistently low hemoglobin levels, Li proposed that urea clearance should be optimized for a better range of Kt/V (Li et al., 2003). Other studies indicated that anemia-related biomarkers, such as the mean corpuscular hemoglobin concentration (MCHC) and transferrin saturation (TS), varied in CKD patients (Gafter-Gvili et al., 2019). As such, it is reasonable to speculate that renal anemia may serve as a promising harbinger of dialysis inadequacy. The medical literature has as of yet failed to reveal sufficient evidence to support this speculation.

To fill this gap in knowledge and produce more references for future studies, we aimed to examine whether anemia-related biomarkers, both individually and as a nomogram, can potentially predict the risk of inadequate PD in patients with CKD.

## Methods

### Study design

The conduct of this study was reviewed and approved by the ethics committees of the Beijing Hospital of Traditional Chinese Medicine, conforming to the principles of the Declaration of Helsinki. All participants provided their informed consent prior to their participation in the study.

### Inclusion criteria

All study participants were Chinese adults who had PD catheter placement and regularly visited the Department of Nephrology at the Beijing Hospital of Traditional Chinese Medicine. This is a retrospective study conducted from September 2017 to October 2022. For consistency, the most recent PD adequacy assessment was recorded from the study participants.

### Exclusion criteria

Initially, 142 patients were recruited in this study, and 15 of them were excluded due to the following reasons: 1) being under hemodialysis or after kidney transplantation; 2) recently experiencing significant changes in their weight; 3) having PD-related peritonitis occurrence in the last 3 months; 4) being diagnosed with diabetic ketoacidosis, acute cardiovascular events, or other severe disorders, including tumors; 5) having no consultation records for 3 months. Finally, 127 eligible patients with complete data were analyzed in this study.

### Demographic information

In addition to the age and gender, the diagnoses of hypertension and diabetes mellitus were also performed at the time of enrollment. Hypertension was defined as having a systolic blood pressure value of ≥140 mmHg, diastolic blood pressure value of ≥90 mmHg, or the current use of antihypertensive agents (Writing Group of 2010 Chinese Guidelines for the Management of Hypertension, 2011). Diabetes mellitus was defined as having a fasting plasma glucose value of ≥7.0 mmol/L or taking hypoglycemic drugs or receiving parenteral insulin therapy (American Diabetes Association, 2013).

### Clinical biomarkers

The collection of clinical biomarkers initiated from October 2022. Venous blood was taken in the fasting state. The total Kt/V value was calculated using the formula recommended in K/DOQI. Hemoglobin (Hgb) and the mean corpuscular hemoglobin concentration were measured using a hematology analyzer. Furthermore, the corresponding reference ranges used for Hgb were 115–150 g/L in women and 120–160 g/L in men. The MCHC was in a range of 316–354 g/L. Percentage of transferrin saturation was calculated by dividing serum iron by the total iron-binding capacity and multiplying the result by 100. TS ranged from 20% to 55%. Creatinine concentrations were determined by the enzymatic method, and urine microalbumin was determined by the immunoturbidimetric method. All assays were conducted at the Clinical Laboratory, Beijing Hospital of Traditional Chinese Medicine.

Based on the ISPD and K/DOQI guidelines, participants were classified into two groups according to total Kt/V, viz., adequate (Kt/V ≥1.7) and inadequate (Kt/V <1.7) groups.

### Statistical analyses

Normally distributed variables are expressed as the mean (standard deviation), skewed variables as the median (interquartile range), and categorical variables as the count (percent). The χ^2^ tests for categorical variables and Wilcoxon rank-sum tests for continuous variables were used to assess the differences in demographic data and clinical biomarkers between the two groups.

To assess the prediction of anemia-related biomarkers with the risk of inadequate PD, logistic regression analysis was performed before and after adjusting for confounders. Gender, age, hypertension, diabetes mellitus, and PD duration were *a priori* balanced in logistic regression analyses. The gender differed significantly between the two groups (*p* = 0.001) in an unadjusted model. Hypertension control was reported as the preserve of the residual kidney function, which had an impact on the calculation of total Kt/V (Mathew et al., 2016). In many studies related to peritoneal dialysis, diabetes mellitus was considered as a factor that may have an influence on the results (Author Anonymous, 1996; Rocco M. et al., 2000; Wang et al., 2003). A younger age and diabetes were reported as significant risk factors for hyperphosphatemia in PD patients (Imtiaz et al., 2017), indicative of inadequate PD. PD, as a form of home dialysis, has a requirement of proficient operation and patience during daily dialysis. As for PD duration, it is reported that the function of the peritoneal membrane and the residual renal function may change rapidly in the first 2 years (Davies et al., 2001), which is the reason why the PD duration was taken into consideration. The effect-size estimate is denoted using odds ratios (ORs) and 95% confidence interval (95% CI).

The calibration capability was assessed using the −2 log likelihood ratio test, the Akaike information criterion (AIC), and the Bayesian information criterion (BIC) to inspect how closely the prediction probability for the addition of significant anemia-related biomarkers reflected the actual observed risk and the global fit of the modified risk model. In addition, decision curve analysis reflects the net benefit of adding anemia-related biomarkers. In this curve, the *X*-axis denotes thresholds for the risk of inadequate PD, and the *Y*-axis denotes net benefits on different thresholds. Furthermore, the area under the receiver operating characteristics curve (AUROC) was also calculated for each model.

The nomogram model was constructed using the “RMS” package in the R programming environment (version 3.5.2), and the predictive accuracy was reflected by the concordance index (C-index). Statistical power was calculated by using power and sample size calculations (PS) software version 3.0.7. Unless otherwise stated, STATA software version 16 (StataCorp, College Station, TX, USA) was used for data cleaning and statistical analyses. Without prior notice, the statistical significance was set at a probability of less than 5%.

## Results

### Baseline characteristics

Table 1 presents the baseline characteristics of the study participants in this study. Of 127 patients involved, 76 were in the adequate group (as controls) and 51 were in the inadequate group (as cases). The gender differed significantly between the two groups (*p* = 0.001). Distributions of age and PD duration, as well as percentages of hypertension and diabetes, were comparable. As for anemia-related biomarkers, only TS was significantly higher in cases than that in controls (*p* = 0.006).

**TABLE 1:** Baseline characteristics of the study participants in the retrospective study.

Characteristic	Overall	Adequate group	Inadequate group	P
Sample size	127	76	51	
Male (n, %)	81 (63.8)	40 (52.6)	41 (80.4)**	0.001
Age (years)	58.26 (13.57)	59.68 (13.10)	56.14 (14.11)	0.122
Diabetes (n, %)	60 (47.2)	34 (44.7)	26 (51.0)	0.491
Hypertension (n, %)	92 (73.0)	52 (69.3)	40 (78.4)	0.261
PD duration (years)	2 [1, 3]	2 [1, 2]	2 [1, 3]	0.106
Kt/V	1.73 [1.57, 1.99]	1.96 [1.84, 2.17]	1.54 [1.44, 1.63]**	<0.001
Scr (µmol/L)	898.20 [727.10, 1,160.25]	829.15 [704.47, 1,006.23]	1,040.50 [806.00, 1,231.80]**	0.001
BUN (mmol/L)	18.77 [15.78, 22.06]	17.45 [14.52, 21.36]	19.56 [16.35, 22.48]*	0.036
Dialysate nitrogen	16.16 (4.94)	15.60 (4.61)	17.05 (5.45)	0.294
Hgb (g/L)	115.50 [108.00, 124.75]	115.50 [108.00, 126.00]	115.00 [104.75, 123.25]	0.545
HCT (%)	34.25 [32.02, 37.27]	34.20 [32.20, 37.62]	34.25 [31.77, 37.10]	0.186
MCV (fL)	90.2 [84.1, 95.2]	90.9 [83.3, 95.7]	89.8 [84.3, 95.6]	0.637
MCH (pg)	30.1 [28.2, 33.2]	29.0 [28.3, 33.4]	30.2 [28.0, 33.1]	0.138
MCHC (g/L)	334.12 (7.93)	334.41 (8.24)	333.69 (7.50)	0.620
TS (%)	26 [19, 32]	28 [22, 32]	22.00 [14, 29]**	0.006
Fer (µg/L)	277 [177, 380]	298.00 [211, 404]	234.00 [172, 360]	0.141

Abbreviations: Kt/V, total Kt/V urea; Scr, serum creatinine; BUN, blood urea nitrogen; Hgb, hemoglobin; HCT, hematocrit; MCV, mean corpuscular volume; MCH, mean corpuscular hemoglobin; MCHC, mean corpuscular hemoglobin concentration; TS, transferrin saturation; Fer, ferritin. Data are expressed as the mean (standard deviation), median (interquartile range), number, or percentage, wherever appropriate.

### Predicting inadequate PD

Table 2 shows the risk prediction of anemia-related biomarkers under study for inadequate PD. After balancing age, gender, hypertension, diabetes mellitus, and PD duration, 20 g/L increment in Hgb was observed to significantly reduce the risk of inadequate PD by 19% (0.81; 0.70 to 0.95; 0.009), 5 g/L increment in the MCHC by 7% (0.93; 0.88 to 0.98; 0.009), and 5% increment in TS by 23% (0.77; 0.64 to 0.94; 0.012). In view of the statistical significance, power estimation revealed that the aforementioned association was convincing, with the power being over 80%.

**TABLE 2:** Risk prediction of three anemia-related biomarkers for inadequate peritoneal dialysis.

Significant risk factor	Odds ratio; 95% confidence interval; P	
	Before adjustment	After adjustment
Hgb (per 20 g/L increment)	0.92, 0.86 to 0.98, 0.014	0.81, 0.70 to 0.95, 0.009
MCHC (per +5 g/L increment)	0.97, 0.96 to 0.99, 0.019	0.93, 0.88 to 0.98, 0.009
TS (per +5% increment)	0.91, 0.83 to 0.99, 0.042	0.77, 0.64 to 0.94, 0.012

Abbreviations: Hgb, hemoglobin; MCHC, mean corpuscular hemoglobin concentration; TS, transferrin saturation; OR, odds ratio; 95% CI, 95% confidence interval.

### Prediction accuracy assessment

**FIGURE 1:**
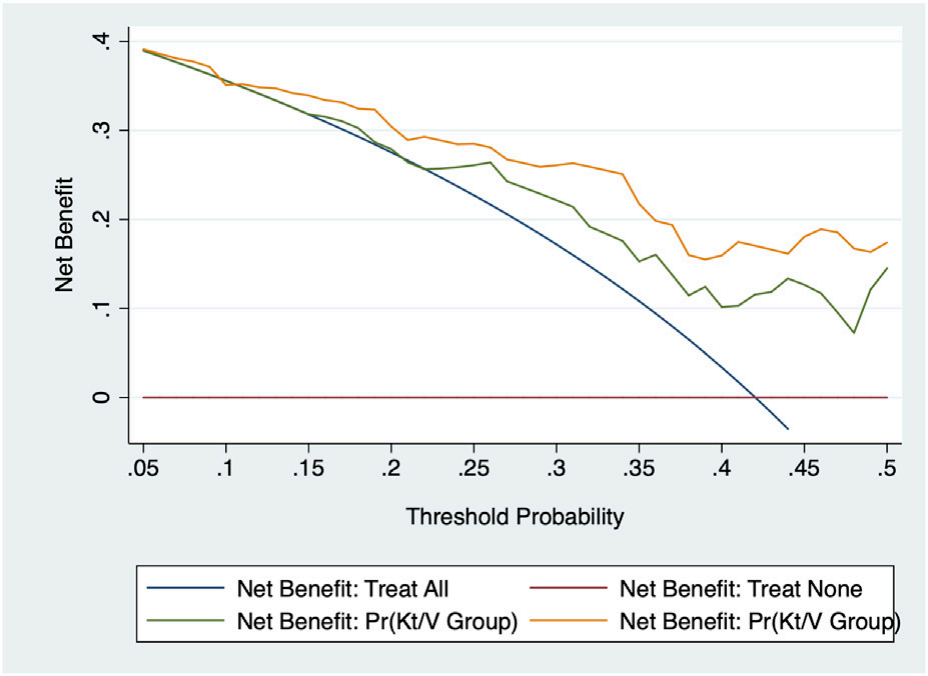
Decision curve analysis of adding three anemia-related biomarkers to the basic model in the prediction of the adequacy of peritoneal dialysis.

Table 3 shows the prediction accuracy driven by adding anemia-related biomarkers to the basic model (including age, gender, diabetes mellitus, hypertension, and PD duration). As for calibration, a reduction in both the AIC and BIC statistics was greater than 10 after adding biomarkers to the basic model. In addition, the likelihood ratio test revealed the significance to be at a level of 3%, which was further confirmed by decision curve analysis (Figure 1) and the AUROC.

**TABLE 3:** Prediction accuracy gained by adding three anemia-related biomarkers to the basic model for inadequate peritoneal dialysis.

Calibration and discrimination statistics	Basic model	Full model
AIC	164.1	82.3
BIC	175.5	97.9
LR test (χ^2^)	14.08	
LR test (P)	0.003	
AUROC (95% CI)	0.819 (0.719; 0.919)	0.719 (0.594; 0.844)
ROC (P)	0.085	

Abbreviations: AIC, Akaike information criterion; BIC, Bayesian information criterion; LR, likelihood ratio. The basic model included age, gender, diabetes mellitus, hypertension, and duration of peritoneal dialysis.

### Nomogram model

**FIGURE 2:**
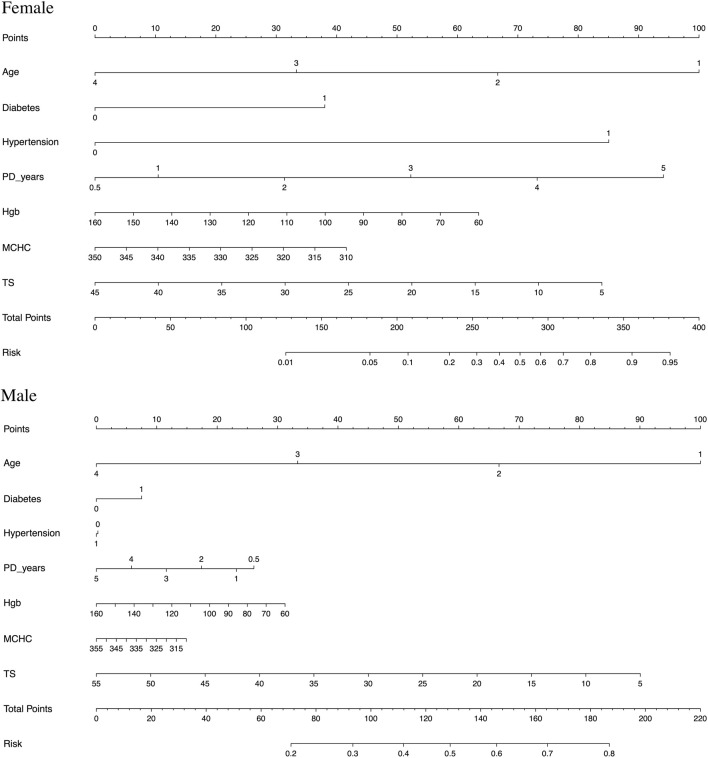
Nomogram prediction models constructed and stratified by gender on the basis of age (age is described as 1–4, with under 30 years old as 1, 31–50 as 2, 51–70 as 3, and 71–90 as 4), diabetes mellitus, hypertension, and PD duration, as well as three significant anemia-related biomarkers (hemoglobin (Hgb), mean corpuscular hemoglobin concentration (MCHC), and transferrin saturation (TS)), respectively.

To facilitate clinical application, a nomogram model was constructed upon being stratified by gender, as illustrated in Figure 2. The prediction model was constructed on the basis of age, diabetes mellitus, hypertension, and PD duration, as well as three significant anemia-related biomarkers. This model was featured by a decent accuracy, as reflected by both the C-index (0.686 for men and 0.825 for women) and calibration curves after bootstrapping 1,000 repetitions (Supplementary Figure S1).

For example, assuming a woman aged 60 years old with diabetes mellitus and hypertension, PD for 3 years, Hgb of 90 g/L, MCHC of 315 g/L, TS of 15%, and her total points being 350, then the probability of experiencing inadequate PD was estimated to be nearly 90%.

## Discussion

The aim of this retrospective study was to examine the prediction of anemia-related biomarkers for inadequate PD in patients with CKD. Our key findings are that the deterioration of three anemia-related biomarkers (Hgb, MCHC, and TS) can precipitate the development of inadequate PD in Chinese patients with CKD. To the best of our knowledge, this is, thus far, the first report that has evaluated the prediction of anemia-related biomarkers for inadequate PD among Chinese adults.

Some studies have examined the association between anemia and PD adequacy, whereas the results of these studies are inconsistent and inconclusive. For instance, a randomized prospective cohort in Hong Kong showed that patients with their total Kt/V maintained below 1.70 had lower levels of Hgb and more severe anemia (Lo et al., 2003). A significant correlation between anemia-related biomarkers and the total Kt/V was demonstrated in a study in Czech Republic, which made a conclusion that the relationship between anemia and blood purification is best expressed using the Kt/V index in PD patients (Opatrny et al., 1999). In addition, data from the Healthcare Financing Administration (HCFA) ESRD Core Indicators Project indicated that people in PD with lower Kt/V tend to have significantly lower mean hematocrit values, with a greater proportion of patients having a hematocrit level of less than 28% (Rocco M. V. et al., 2000). A retrospective cohort study in China demonstrated that PD patients with dialysis inadequacy had lower Hgb levels (Li et al., 2003). However, a study from Canada reported that Kt/V provided by PD did not seem important in the improvement of anemia (Ossareh et al., 2003). The reasons behind these conflicting findings are manifold. The first reason might be due to the differences in study populations. Mounting evidence suggests that inter-individual differences in anemia parameters have been linked in part to genetic polymorphisms in dialysis patients (Girndt et al., 2006; Kopp and Winkler, 2018). The second reason might be related to unaccounted residual confounding, which might yield a possible selection bias. The third reason is that the contribution of any individual biomarkers to reflect the level of Kt/V and the adequacy of PD is likely to be small, considering the complex nature of PD (Li and Szeto, 2003; Ryckelynck et al., 2012).

The important finding of this study is that TS differed significantly between patients in the adequate and inadequate groups, which is rarely reported in the medical literature. This finding is biologically plausible. TS can reflect a soluble transferrin receptor, which is a biomarker of erythropoiesis and is often impaired in dialysis patients (Gafter-Gvili et al., 2019). There is also evidence that TS can reflect the levels of anemia in PD patients and can further indicate the inadequacy of dialysis (Yavuz et al., 2004; Chung et al., 2012; Belo et al., 2019). Moreover, further regression analyses revealed that an increase in Hgb, MCHC, and TS levels was independently and significantly associated with the reduced risk of inadequate PD in patients with CKD. In light of the cross-sectional nature of this study, our findings support the candidacy of anemia-related biomarkers in the development of inadequate PD among patients with CKD.

Because of the complicated profiles of inadequate PD in patients with CKD, the contribution of individual biomarkers may be small or dependent on the presence of other biomarkers. The majority of studies in this field focused on single biomarkers, while disregarding other relevant biomarkers and overlooking their joint contribution. In this study, we not only assessed the association of anemia-related biomarkers with inadequate PD individually but also jointly in the form of a nomogram. In view of the marked gender differences in the incidence of inadequate PD among patients with CKD, we separately constructed nomogram models for anemia-related biomarkers and the risk of substandard Kt/V by gender, and both models were featured through decent prediction accuracy. For practical reasons, these nomogram models can be routinely applied in clinical practice to facilitate clinical decision-making and the personalized management of PD.

Several possible limitations should be acknowledged for this study. First, the cross-sectional data on this study preclude further comments on the cause–effect relationship between anemia-related biomarkers and Kt/V, and all study participants were recruited from one center, requiring further external validation. Second, some unmeasured characteristics of study subjects, such as dietary habits, might confound the association of anemia-related biomarkers with PD (Finkelstein et al., 2011). Third, all study participants were enrolled from a single center, which might yield a possibility of population stratification and limit the extrapolation of our conclusions. We agree that future studies that examined the implication of anemia-related biomarkers in inadequate PD are encouraged.

In spite of these limitations, our findings indicate the deterioration of three anemia-related biomarkers (Hgb, MCHC, and TS) can precipitate the development of inadequate PD in Chinese patients with CKD. Importantly, we have constructed a nomogram model based on anemia-related biomarkers for predicting inadequate PD, which can help provide evidence for medical decision-making and arguments for future research work on anemia as candidate monitors. Meanwhile, we hope that further investigations on the molecular mechanisms linking anemia-related biomarkers and PD inadequacy are warranted.

## Data Availability

The raw data supporting the conclusion of this article will be made available by the authors, without undue reservation.
